# Membrane Cholesterol Interactions with Proteins in Hypercholesterolemia-Induced Endothelial Dysfunction

**DOI:** 10.1007/s11883-023-01127-w

**Published:** 2023-07-07

**Authors:** Ibra S. Fancher, Irena Levitan

**Affiliations:** 1 Department of Kinesiology and Applied Physiology, College of Health Sciences, University of Delaware, Newark, DE USA; 2 Division of Pulmonary, Critical Care, Sleep and Allergy, Department of Medicine, College of Medicine, University of Illinois at Chicago, Chicago, IL USA

**Keywords:** Cholesterol, Kir2.1, Hypercholesterolemia, Dyslipidemia, Atherosclerosis, Endothelial dysfunction

## Abstract

**Purpose of Review:**

The goal of this review is to highlight work identifying mechanisms driving hypercholesterolemia-mediated endothelial dysfunction. We specifically focus on cholesterol-protein interactions and address specific questions related to the impact of hypercholesterolemia on cellular cholesterol and vascular endothelial function. We describe key approaches used to determine the effects of cholesterol-protein interactions in mediating endothelial dysfunction under dyslipidemic conditions.

**Recent Findings:**

The benefits of removing the cholesterol surplus on endothelial function in models of hypercholesterolemia is clear. However, specific mechanisms driving cholesterol-induced endothelial dysfunction need to be determined. In this review, we detail the latest findings describing cholesterol-mediated endothelial dysfunction, highlighting our studies indicating that cholesterol suppresses endothelial Kir2.1 channels as a major underlying mechanism.

**Summary:**

The findings detailed in this review support the targeting of cholesterol-induced suppression of proteins in restoring endothelial function in dyslipidemic conditions. The identification of similar mechanisms regarding other cholesterol-endothelial protein interactions is warranted.

## Introduction

Plasma hypercholesterolemia, elevated levels of low-density lipoproteins (LDL), is one of the major predictors and risk factors for the development of atherosclerosis. The mechanisms responsible for the atherogenic effects of LDL are primarily attributed to the penetration and retention of LDL particles in the extracellular sub-endothelium space, where they undergo pro-inflammatory modifications by aggregation and oxidation and induce recruitment of monocytes that differentiate into macrophages to become cholesterol-laden foam cells [1, 2]. Accumulation of cholesterol can be in the form of cholesterol esters that can be stored intracellularly or non-esterified (free) cholesterol, an essential and major lipid component of the plasma membrane in all mammalian cells, where it exerts numerous effects on the function of membrane proteins both directly and indirectly [3–5]. Earlier studies found a strong correlation between free cholesterol content of the lesions and lesion necrosis due to cholesterol toxicity leading to macrophage death [6, 7]. The toxicity of cholesterol was attributed to a change in the biophysical properties of the ER membranes and ER stress [8] and cholesterol-induced interaction with membrane receptors [9]. A key early stage of atherosclerosis development, however, is impairment of the endothelium, the inner lining of the blood vessels. In this review, we focus on the accumulating evidence that hypercholesterolemia-induced increase in the membrane cholesterol content of endothelial cells plays a major role in endothelial dysfunction via direct cholesterol-protein interactions.

## Impact of Plasma Hypercholesterolemia on Endothelial Free Cholesterol

The first key question is what is the impact of plasma hypercholesterolemia on cellular and membrane cholesterol content of endothelial cells? In general, membrane cholesterol of mammalian cells is maintained within a relatively narrow range of a physiological set point regulated by sterol sensing feedback mechanisms [10–12]. Numerous studies also showed, however, that cholesterol content of cellular membranes in cultured cells, including endothelial cells, can be increased ~ twofold by the exposure to a cyclic oligosaccharide methyl-β-cyclodextrin (MβCD) saturated with free cholesterol [13]. Surprisingly, while endothelial dysfunction is well recognized to be a key early step in hypercholesterolemia-induced vascular dysfunction and initiation of atherosclerosis, only few studies assessed the level of free cholesterol in endothelial cells under hypercholesterolemic conditions. First, our studies showed that plasma hypercholesterolemia in vivo [14] and exposure to high levels of LDL in vitro [15, 16••] both result in significant elevation of free cholesterol in endothelial cells. Specifically, using a porcine model of diet-induced hypercholesterolemia, we showed that aortic endothelial cells freshly isolated from hypercholesterolemic pigs contained a twofold higher free cholesterol level, as compared to cells isolated from aortas of pigs that were maintained on regular low-cholesterol diet (~ 20 μg cholesterol/mg protein vs. ~ 40 μg cholesterol/mg protein) [14]. A similar elevation of free cholesterol was observed when human aortic endothelial cells were exposed to VLDL or to acLDL but not to oxLDL, the latter having no effect on endothelial cellular cholesterol [14]. In this early study, we focused on the modified forms of LDL rather than on LDL itself because of the previous extensive literature showing that exposure to LDL does not result in significant cholesterol loading of macrophages, whereas acLDL does giving rise to the hypothesis that the major source of cholesterol loading in vivo is oxLDL [17, 18]. The rationale to infer observations obtained using acLDL to oxidative modifications of LDL that occur in vivo was that both forms of the modified LDL are recognized and internalized by the same scavenger receptors [17]. However, a more detailed comparison of the actual impacts of acLDL and oxLDL on cellular cholesterol revealed that, at least in the endothelial cells, it is completely different: while exposure to the acLDL indeed loads endothelial cells with cholesterol, exposure to oxLDL does not but instead loads the cells with oxidized lipids, primarily oxysterols [19, 20]. Oxysterols also incorporate into the plasma membrane but their effect on membrane structure is the opposite of that of cholesterol: while cholesterol increases lipid order of the membrane, oxysterols disrupt lipid order [21, 22]. Recently, we showed that this is also true in macrophages, exposure of bone marrow-derived macrophages to oxLDL resulted in loading the cells with 7ketocholesterol but not cholesterol [23]. Thus, we next determined if exposure to physiological levels of LDL may result in cholesterol loading of endothelial cells and found that this is indeed the case. Using mass spectrometry, we found that exposing human aortic endothelial cells to the LDL level observed in hypercholesterolemic patients (250 mg/dl) resulted in a significant increase in cholesterol/phospholipid ratio in the membrane, as compared to cells exposed to 50 mg/dl LDL, a level found in healthy individuals (~ 600 to ~ 750 pmol cholesterol/nmol phospholipid, respectively) [15]. This elevation of cholesterol was sufficient to result in a significant ordering of the membrane, as assessed by a Laurdan dye [15], sensitive to the water dipoles in the membrane [24, 25]. Most recently, we found that exposure to increasing levels of LDL results in a gradual increase in endothelial free cholesterol with a slight but statistically significant increase in response to 150 mg/dl LDL and a relatively large twofold increase/per mg protein, in response to 250 mg/dl LDL [16••]. It is noteworthy that exposing endothelial cells to MβCD-cholesterol, high levels of LDL and to diet-induced hypercholesterolemia in vivo, all resulted in ~ twofold increase in cellular free cholesterol when normalized to protein, suggesting that this is the amount that cellular membranes can accommodate when exposed to hypercholesterolemic conditions.

## Impact of Cholesterol Loading on Endothelial Function In Vitro

The next logical question is what is the impact of cholesterol loading on endothelial function? This question can be addressed in vitro by modulating cellular cholesterol level pharmacologically, particularly using MβCD, which provides a highly reproducible method to both deplete and enrich cells with cholesterol. However, depletion of cellular cholesterol, while definitely uncovering fundamental roles of cholesterol in cellular function, does not necessarily provide significant insights to the physiologically relevant conditions of cholesterol loading. Indeed, several examples were reported when cholesterol depletion had an effect on specific features/functions, whereas cholesterol enrichment had no effect [26]. We focus here, therefore, on discussing endothelial dysfunction induced specifically by cholesterol enrichment.

An early work by Feron et al. [27] showed that exposing endothelial cells to serum obtained from hypercholesterolemic patients resulted in an increase in cellular abundance of a cholesterol binding protein, caveolin-1, a structural scaffolding protein of membrane invaginations called caveolae, known to be signaling hubs [28]. Caveolin-1 is also known to directly interact and regulate the function of multiple proteins, including negative regulation of endothelial nitric synthase, eNOS, an enzyme that produces NO [28]. Feron et al. also showed that an increase in caveolin-1 led to stabilization of caveolin-1-eNOS interaction and a decrease in eNOS function. However, even though caveolae constitute a membrane domain strongly enriched in cholesterol, it is not entirely clear whether an increase in the abundance of caveolin-1 was a result of an increase in membrane cholesterol. Our studies demonstrated a direct inhibitory effect of free cholesterol loading on endothelial inwardly rectifying K + channels (Kir2.1) [14, 29], a flow-sensitive channel that has been proposed to be one of the putative endothelial flow sensors [30]. Furthermore, our extensive computational and structure–function studies revealed that cholesterol suppresses Kir channels via specific cholesterol-protein interactions [31, 32] with the cholesterol molecules binding to multiple hydrophobic pockets of the channel transmembrane domains constituting non-annular cholesterol binding sites [33, 34]. Notably cholesterol-induced suppression of Kir channels was shown to be independent of caveolin [35]. We further discovered that the mechanism by which cholesterol binding suppresses the activity of Kir2 channels is disrupting specific intra-molecule residue-residue interaction that leads to the uncoupling of the channel subunits [36•], which interferes with the channel gating [37, 38]. In terms of the functional consequences of cholesterol-induced suppression of endothelial Kir channels, since functional expression of these channels is essential for the activation of a major flow-induced phosphorylation cascade of Akt1/eNOS [39], their cholesterol suppression is expected to impair flow-induced production of NO. Additionally, Andrews et al. showed that cholesterol loading suppresses ATP-induced-induced capacitative Ca^2+^ entry, indicating a decreased function of the STIM/ORAI complex that constitutes the capacitative Ca^2+^ entry mechanism [40], which was accompanied with an inhibition of eNOS phosphorylation. Since capacitative Ca^2+^ entry was shown earlier to contribute to agonist- and shear-stress activation of eNOS [41], the authors suggested that cholesterol-induced impairment of Ca^2+^ entry might be responsible for the inhibition of eNOS. All these studies, however, focus on acute effects of free cholesterol loading on different endothelial proteins, and clearly complementary in vivo studies are needed to establish which of these mechanisms contribute to hypercholesterolemia-induced endothelial dysfunction.

## Impact on Endothelial Function Following the Removal of Cholesterol Surplus from Hypercholesterolemic Animals

The beneficial effects of increased HDL and cholesterol-lowering therapies (e.g., statins) on endothelial function indicates that the removal of excess free cholesterol from endothelial membranes improves endothelial function in individuals with hypercholesterolemia [42, 43]. Therefore, another approach to determine the role of excess cellular cholesterol on endothelial function is by experimentally removing the surplus of cholesterol from hypercholesterolemic animals in vivo and from tissue ex vivo. In this approach, the removal of excess cholesterol is predicted to restore the proper endothelial functions. For instance, Kaul et al. showed that in vivo infusions of a naturally occurring and antiatherosclerotic apolipoprotein variant established to have increased cholesterol efflux capacity reversed endothelial dysfunction in aortas of dyslipidemic apolipoprotein E deficient (*Apoe*^−/−^) mice [44]. *Apoe*^*−/−*^ mice receiving the apolipoprotein variant had significantly less aortic tissue cholesterol compared to control mice which corresponded to improved endothelial function. Importantly, these effects were independent of serum cholesterol which remained elevated in *Apoe*^*−/−*^ mice receiving the apolipoprotein variant suggesting that the removal of tissue cholesterol specifically restores endothelial function [44]. However, these earlier studies did not mechanistically address how the removal of the cholesterol surplus from vascular tissue improved endothelial function nor was it determined if indirect systemic effects of the apolipoprotein afforded benefits to endothelial function in vivo. Therefore, we aimed to determine if the beneficial effect of the removal of a cholesterol surplus ex vivo in arteries from *Apoe*^*−/−*^ mice was mediated by restoration of inwardly rectifying K^+^ (Kir) channels. To achieve this goal, we exposed isolated mesenteric arteries to MβCD and performed pressure myography to assess endothelial function. As expected, arteries from *Apoe*^*−/−*^ mice had blunted dilations to flow, a potent stimulus that induces NO via endothelial Kir channel activation [39, 45•]. In contrast, arteries from *Apoe*^*−/−*^ mice exposed to MβCD prior to testing endothelial function exhibited comparable dilations to flow as that of arteries from WT mice. Indeed, Kir channel function was restored in this condition as Ba^2+^, an inhibitor of Kir channels, blunted the dilatory response to flow indicating that the removal of excess cholesterol from arteries of dyslipidemic mice restored endothelial function via recovery of Kir channel function. Furthermore, MβCD-induced recovery of the flow-induced dilation was abrogated in arteries of *Apoe*^*−/−*^ mice crossed with Kir2.1-deficient mice, indicating that the recovery of the dilations is Kir2.1 dependent. This finding was further supported by patch clamp electrophysiology experiments in freshly isolated endothelial cells from *Apoe*^*−/−*^ mice where MβCD treatment resulted in a significant increase in Kir channel current [45•]. Taken together, these observations indicate that the removal of the cholesterol surplus in vivo or ex vivo has beneficial effects on endothelial function, likely through the restoration of Kir channel function upstream of NO production. However, the pan removal of excess cholesterol by promoting cholesterol efflux undoubtedly has multiple possible effects on endothelial function. To best address a specific contribution of Kir to cholesterol-induced endothelial dysfunction, we must specifically disrupt the ability of cholesterol to suppress Kir channels without reducing free cholesterol in the membrane.

## Benefits to Endothelial Function by Rendering Proteins Insensitive to Cholesterol

**Fig. 1:**
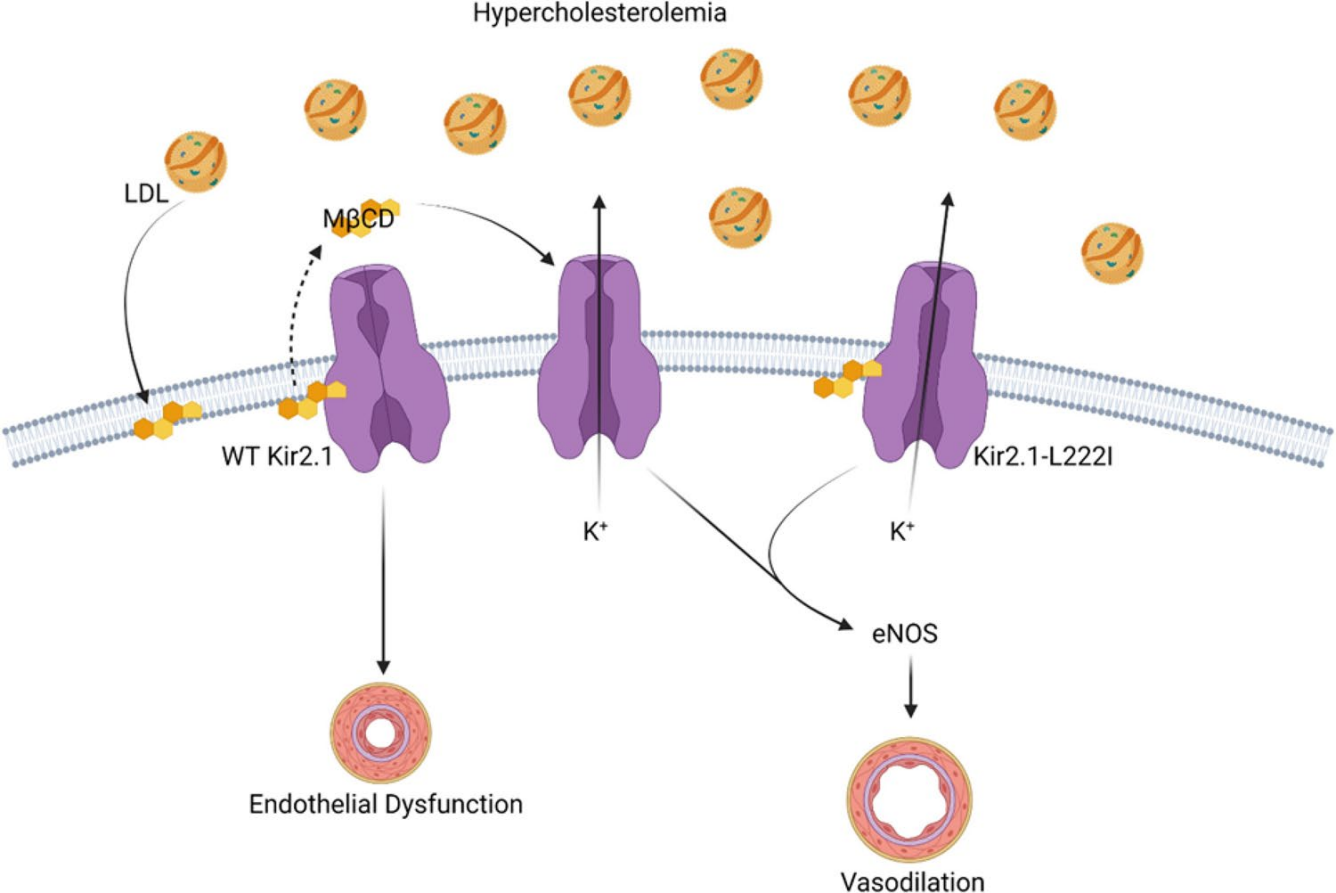
Schematic detailing restoration of endothelial function via reversing/preventing cholesterol-mediated suppression of Kir channels. By removing the surplus of cholesterol using methyl-β-cyclodextrin (MβCD) or preventing cholesterol-mediated suppression of endothelial Kir channels (L222I mutation), we were able to restore endothelial function in a model of hypercholesterolemia. It is important to note that the L222I mutation does not disrupt the interaction between cholesterol and Kir2.1 but renders the channel insensitive to cholesterol-mediated suppression [46]. Figure created with BioRender.com

The most direct approach in determining if cholesterol mediates endothelial dysfunction via direct effects on the function of a specific protein is to generate functional mutants that are insensitive to cholesterol-mediated suppression. In this approach, the cholesterol surplus accompanying dyslipidemia is maintained and only the role of the cholesterol-mediated suppression of the protein in inducing endothelial dysfunction is addressed. With regard to our findings detailing cholesterol-mediated suppression of endothelial Kir2.1 channels, our structure–function studies described above led to the identification of an array of mutations that (i) did not affect Kir channel function and (ii) rendered Kir channels insensitive to cholesterol-mediated suppression of channel function. We previously identified that the Kir2.1-L222I mutation matched these criteria [46]. Our next major goal was to determine if this cholesterol insensitive Kir2.1 mutant could restore endothelial function in a model of hypercholesterolemia. We generated Kir2.1-L222I transgenic mice using CRSPR-Cas9 gene editing and crossed them with *Apoe*^*−/−*^ mice. We first tested Kir channel currents in freshly isolated endothelial cells to confirm that the Kir2.1-L222I channels had (i) larger currents than *Apoe*^*−/−*^ controls containing WT Kir2.1 and (ii) restored flow-sensitivity. Indeed, endothelial cells from the Kir2.1-L222I *Apoe*^*−/−*^ transgenic mice had larger Kir currents than the WT Kir2.1 *Apoe*^*−/−*^ controls. Furthermore, in contrast to WT Kir2.1 channels in cells from *Apoe*^*−/−*^ mice, the Kir2.1-L222I channels exhibited flow-sensitivity indicating a complete restoration of function in the cholesterol-insensitive mutant channels [16••]. Most importantly, we next aimed to determine if rendering Kir channels insensitive to cholesterol prevented the dyslipidemia-induced endothelial dysfunction observed in WT Kir2.1 *Apoe*^*−/−*^ controls. Arteries from the mice containing the Kir2.1-L222I mutation exhibited dilations to flow comparable to non-dyslipidemic, WT mice independent of the presence of dyslipidemia indicating that rendering Kir2.1 insensitive to cholesterol-mediated suppression was sufficient to restore endothelial function without removing the cholesterol surplus [16••]. Our findings, summarized in Fig. 1, support the targeting of cholesterol-induced suppression of proteins in restoring endothelial function in dyslipidemic conditions and promote the identification of similar mechanisms in distinct endothelial proteins.

## Conclusion

The beneficial effects on endothelial function following the removal of the cholesterol surplus under hypercholesterolemic conditions are clear. However, we are just beginning to unveil the mechanisms related to specific cholesterol-protein interactions in mediating endothelial dysfunction. Our recent studies have detailed that the cholesterol-mediated suppression of Kir2.1 is a major and specific mechanism underlying hypercholesterolemia-induced endothelial dysfunction. Importantly, rendering this channel insensitive to cholesterol-mediated suppression of channel function restores endothelial function in the presence of elevated membrane cholesterol, thereby highlighting the potential benefits of targeting the effects of cholesterol on specific proteins. These findings may lead to an additional avenue in restoring endothelial function in dyslipidemic populations, perhaps in combination with lipid lowering therapies [47].

## References

[1] Tabas I, Williams KJ, Boren J (2007). Subendothelial lipoprotein retention as the initiating process in atherosclerosis: update and therapeutic implications. Circulation.

[2] Nakashima Y, Fujii H, Sumiyoshi S, Wight TN, Sueishi K (2007). Early human atherosclerosis: accumulation of lipid and proteoglycans in intimal thickenings followed by macrophage infiltration. Arterioscler Thromb Vasc Biol.

[3] Yeagle PL. Modulation of membrane function by cholesterol. Biochimie. 1991;73:1303. 10.1016/0300-9084(91)90093-g. 10.1016/0300-9084(91)90093-g 1664240

[4] Yeagle PL. Non-covalent binding of membrane lipids to membrane proteins. Biochim Biophys Acta (BBA) - Biomembr. 2013(0). 10.1016/j.bbamem.2013.11.009. 10.1016/j.bbamem.2013.11.009 24269542

[5] Zakany F, Kovacs T, Panyi G, Varga Z (2020). Direct and indirect cholesterol effects on membrane proteins with special focus on potassium channels. Biochim Biophys Acta Mol Cell Biol Lipids.

[6] Ball RY, Stowers EC, Burton JH, Cary NR, Skepper JN, Mitchinson MJ (1995). Evidence that the death of macrophage foam cells contributes to the lipid core of atheroma. Atherosclerosis.

[7] Libby P, Geng YJ, Aikawa M, Schoenbeck U, Mach F, Clinton SK (1996). Macrophages and atherosclerotic plaque stability. Curr Opin Lipidol.

[8] Devries-Seimon T, Li Y, Yao PM, Stone E, Wang Y, Davis RJ (2005). Cholesterol-induced macrophage apoptosis requires ER stress pathways and engagement of the type A scavenger receptor. J Cell Biol.

[9] Sun Y, Ishibashi M, Seimon T, Lee M, Sharma SM, Fitzgerald KA (2009). Free cholesterol accumulation in macrophage membranes activates Toll-like receptors and p38 mitogen-activated protein kinase and induces cathepsin K. Circ Res.

[10] Fielding CJ, Fielding PE (1995). Role of an N-ethylmaleimide-sensitive factor in the selective cellular uptake of low-density lipoprotein free cholesterol. Biochemistry.

[11] Luo J, Yang H, Song BL (2020). Mechanisms and regulation of cholesterol homeostasis. Nat Rev Mol Cell Biol.

[12] Steck TL, Tabei SMA, Lange Y (2021). A basic model for cell cholesterol homeostasis. Traffic.

[13] Zidovetzki R, Levitan I (2007). Use of cyclodextrins to manipulate plasma membrane cholesterol content: evidence, misconceptions and control strategies. Biochim BiophysActa (BBA) - Biomembr.

[14] Fang Y, Mohler ER, Hsieh E, Osman H, Hashemi SM, Davies PF (2006). Hypercholesterolemia suppresses inwardly rectifying K+ channels in aortic endothelium in vitro and in vivo. Circ Res.

[15] Bogachkov YY, Chen L, Le Master E, Fancher IS, Zhao Y, Aguilar V (2020). LDL induces cholesterol loading and inhibits endothelial proliferation and angiogenesis in Matrigels: correlation with impaired angiogenesis during wound healing. Am J Physiol Cell Physiol.

[16] Ahn SJ, Fancher IS, Granados ST, Do Couto NF, Hwang CL, Phillips SA (2022). Cholesterol-induced suppression of endothelial Kir channels is a driver of impairment of arteriolar flow-induced vasodilation in humans. Hypertension.

[17] Levitan I, Volkov S, Subbaiah PV (2010). Oxidized LDL: diversity, patterns of recognition, and pathophysiology. Antioxid Redox Signal.

[18] Steinberg D, Parthasarathy S, Carew TE, Khoo JC, Witztum JL (1989). Beyond cholesterol. Modifications of low-density lipoprotein that increase its atherogenicity. N Engl J Med.

[19] Byfield FJ, Tikku S, Rothblat GH, Gooch KJ, Levitan I (2006). OxLDL increases endothelial stiffness, force generation and network formation. J Lipid Res.

[20] Shentu TP, Titushkin I, Singh DK, Gooch KJ, Subbaiah PV, Cho M (2010). oxLDL-induced decrease in lipid order of membrane domains is inversely correlated with endothelial stiffness and network formation. Am J Physiol Cell Physiol.

[21] Shentu TP, Singh DK, Oh M-J, Sun S, Sadaat L, Makino A (2012). The role of oxysterols in control of endothelial stiffness. J Lipid Res.

[22] Ayee MAA, Levitan I (2021). Lipoprotein-induced increases in cholesterol and 7-ketocholesterol result in opposite molecular-scale biophysical effects on membrane structure. Front Cardiovasc Med.

[23] Rezende L, Couto NFD, Fernandes-Braga W, Epshtein Y, Alvarez-Leite JI, Levitan I (2022). OxLDL induces membrane structure rearrangement leading to biomechanics alteration and migration deficiency in macrophage. Biochim Biophys Acta Biomembr.

[24] Gaus K, Zech T, Harder T (2006). Visualizing membrane microdomains by Laurdan 2-photon microscopy. Mol Membr Biol.

[25] Levitan I (2021). Evaluating membrane structure by Laurdan imaging: disruption of lipid packing by oxidized lipids. Curr Top Membr.

[26] Byfield F, Aranda-Aspinoza H, Romanenko VG, Rothblat GH, Levitan I (2004). Cholesterol depletion increases membrane stiffness of aortic endothelial cells. Biophys J.

[27] Feron O, Dessy C, Moniotte S, Desager JP, Balligand JL (1999). Hypercholesterolemia decreases nitric oxide production by promoting the interaction of caveolin and endothelial nitric oxide synthase. J Clin Invest.

[28] Minshall RD, Sessa WC, Stan RV, Anderson RG, Malik AB (2003). Caveolin regulation of endothelial function. Am J Physiol Lung Cell Mol Physiol.

[29] Romanenko VG, Rothblat GH, Levitan I (2002). Modulation of endothelial inward-rectifier K+ current by optical isomers of cholesterol. Biophys J.

[30] Olesen SP, Clapham DE, Davies PF (1988). Haemodynamic shear stress activates a K+ current in vascular endothelial cells. Nature.

[31] Singh DK, Shentu TP, Enkvetchakul D, Levitan I (2011). Cholesterol regulates prokaryotic Kir channel by direct binding to channel protein. Biochim Biophys Acta.

[32] Rosenhouse-Dantsker A, Noskov S, Han H, Adney SK, Tang QY, Rodriguez-Menchaca AA (2012). Distant cytosolic residues mediate a two-way molecular switch that controls the modulation of inwardly rectifying potassium (Kir) channels by cholesterol and phosphatidylinositol 4,5-bisphosphate (PI(4,5)P(2)). J Biol Chem.

[33] Rosenhouse-Dantsker A, Noskov S, Durdagi S, Logothetis DE, Levitan I (2013). Identification of novel cholesterol-binding regions in Kir2 channels. J Biol Chem.

[34] Barbera N, Ayee MAA, Akpa BS, Levitan I (2018). Molecular dynamics simulations of Kir2.2 interactions with an ensemble of cholesterol molecules. Biophys J.

[35] Han H, Rosenhouse-Dantsker A, Gnanasambandam R, Epshtein Y, Chen Z, Sachs F (2014). Silencing of Kir2 channels by caveolin-1: cross-talk with cholesterol. J Physiol.

[36] Barbera N, Granados ST, Vanoye CG, Abramova TV, Kulbak D, Ahn SJ (2022). Cholesterol-induced suppression of Kir2 channels is mediated by decoupling at the inter-subunit interfaces. iScience.

[37] Wang S, Vafabakhsh R, Borschel WF, Ha T, Nichols CG (2016). Structural dynamics of potassium-channel gating revealed by single-molecule FRET. Nat Struct Mol Biol.

[38] Borschel WF, Wang S, Lee S, Nichols CG (2017). Control of Kir channel gating by cytoplasmic domain interface interactions. J Gen Physiol.

[39] Ahn SJ, Fancher IS, Bian JT, Zhang CX, Schwab S, Gaffin R (2017). Inwardly rectifying K(+) channels are major contributors to flow-induced vasodilatation in resistance arteries. J Physiol.

[40] Andrews AM, Muzorewa TT, Zaccheo KA, Buerk DG, Jaron D, Barbee KA (2017). Cholesterol enrichment impairs capacitative calcium entry, eNOS phosphorylation & shear stress-induced NO production. Cell Mol Bioeng.

[41] Andrews AM, Jaron D, Buerk DG, Barbee KA (2014). Shear stress-induced NO production is dependent on ATP autocrine signaling and capacitative calcium entry. Cell Mol Bioeng.

[42] Tran-Dinh A, Diallo D, Delbosc S, Varela-Perez LM, Dang QB, Lapergue B (2013). HDL and endothelial protection. Br J Pharmacol.

[43] Wolfrum S, Jensen KS, Liao JK (2003). Endothelium-dependent effects of statins. Arterioscler Thromb Vasc Biol.

[44] Kaul S, Coin B, Hedayiti A, Yano J, Cercek B, Chyu KY (2004). Rapid reversal of endothelial dysfunction in hypercholesterolemic apolipoprotein E-null mice by recombinant apolipoprotein A-I(Milano)-phospholipid complex. J Am Coll Cardiol.

[45] • Fancher IS, Ahn SJ, Adamos C, Osborn C, Oh MJ, Fang Y, et al. Hypercholesterolemia-induced loss of flow-induced vasodilation and lesion formation in apolipoprotein E-deficient mice critically depend on inwardly rectifying K(+) channels. J Am Heart Assoc. 2018;7(5):e007430. 10.1161/JAHA.117.007430. **In this study, the authors show that cholesterol-induced suppression of endothelial Kir channels plays a key role in the impairment of endothelial function in a mouse model of hypercholesterolemia.** 10.1161/JAHA.117.007430 PMC5866319 29502106

[46] Rosenhouse-Dantsker A, Noskov S, Logothetis DE, Levitan I (2013). Cholesterol sensitivity of KIR2.1 depends on functional inter-links between the N and C termini. Channels (Austin).

[47] Hudgins EC, Bonar AM, Nguyen T, Fancher IS (2022). Targeting lipid-ion channel interactions in cardiovascular disease. Front Cardiovasc Med.

